# Stimulating Sunscreen Use Among Outdoor Construction Workers: A Pilot Study

**DOI:** 10.3389/fpubh.2022.857553

**Published:** 2022-04-01

**Authors:** Anne J. Keurentjes, Sanja Kezic, Thomas Rustemeyer, Carel T. J. Hulshof, Henk F. van der Molen

**Affiliations:** ^1^Department of Public and Occupational Health, Amsterdam Public Health Research Institute, Amsterdam UMC, University of Amsterdam, Amsterdam, Netherlands; ^2^Department of Dermatology and Allergology, Amsterdam University Medical Centers (UMC), Amsterdam, Netherlands

**Keywords:** outdoor workers, solar radiation, non-melanoma skin cancer, sunscreen use, occupational disease, ultraviolet exposure, stratum corneum, biomarkers

## Abstract

**Background:**

Outdoor workers (OW) receive a higher dose of ultraviolet radiation (UVR) compared to indoor workers (IW) which increases the risk of non-melanoma skin cancer (NMSC). Regular sunscreen use reduces the NMSC risk, however, adequate sun-safety behavior among OW is poor. The main objective was to conduct method- and intervention-related elements of a future intervention trial among OW, based on providing sunscreen and assessing sunscreen use on group- and individual level.

**Methods:**

This pilot study was conducted at a construction site in the Netherlands from May-August 2021. Nine dispensers with sunscreen (SPF 50+) were installed at the worksite. OW (*n* = 67) were invited to complete two (cross-sectional) questionnaires on sun-safety behavior, before and after providing sunscreen. Stratum corneum (SC) samples for the assessment of UV-biomarkers were collected from the forehead and behind the ear from 15 OW and 15 IW. The feasibility of the following elements was investigated: recruitment, (loss to) follow-up, outcome measures, data collection, and acceptability of the intervention.

**Results:**

The first questionnaire was completed by 27 OW, the second by 17 OW. More than 75 percent of the OW were aware of the risks of sun exposure, and 63% (*n* = 17) found sunscreen use during worktime important. The proportion of OW never applying sunscreen in the past month was 44.4% (*n* = 12) before, and 35.3% (*n* = 6) after providing sunscreen. A majority of OW (59.3%, *n* = 16) found sunscreen provision encouraging for sunscreen use, the dispensers easy to use (64.7%, *n* = 17) and placed in practical spots (58.8%, *n* = 18). Collecting SC-samples was fast and easy, and several UV-biomarkers showed higher levels for sun-exposed compared to less exposed body parts. There was no significant difference in UV-biomarker levels between OW and IW.

**Conclusions:**

This pilot study revealed low sunscreen use among OW despite providing sunscreen, overall satisfaction with the sunscreen, and the sufficient awareness of the risks of UVR-exposure. Collecting SC-samples at the workplace is feasible and several UV-biomarkers showed to be promising in assessing UVR-exposure. The low participation rate and high loss to follow-up poses a challenge for future intervention studies.

## Introduction

Non-melanoma skin cancer (NMSC) incidence is rising in outdoor workers (OW) (1). The main cause of NMSC is exposure to solar ultraviolet radiation (UVR) and occupational exposure contributes to the overall lifetime UV dose (2, 3). The high and increasing incidence rates of NMSC—including frequent recurrence—have a considerable impact on the quality of life of the affected workers, and pose a significant burden for the health care system (4). The association between occupational UVR exposure and NMSC prevalence is recognized by the World Health Organization (WHO) and the International Labour Organization (ILO) (5, 6), and in six EU countries NMSC is also listed as an occupational disease (7).

NMSC can be avoided, if adequate measures to reduce UVR exposure are taken. There are several possible prevention strategies, including sunscreen use (8). Sunscreen is shown to be an effective strategy to reduce UVR exposure and its health consequences (9, 10). It is reported as a feasible measure to adopt by OW (11–13), and with regular use, sunscreens are able to prevent the formation of skin (pre)malignancies (9, 10). However, previous research revealed several barriers for OW to use sunscreen. These include the common belief that people with a tanned or dark skin are not at risk for skin cancer and protective measures are not necessary (9, 14), or that applying sunscreen is seen as a disturbance and a nuisance (9, 15, 16). Many OW are male and some feel it is not masculine to protect themselves from the sun (9, 17, 18). Adequate sun-safety behavior among outdoor workers is still poor (9, 19, 20), with examples of OW never using sunscreen and reporting sunburns during worktime (9). An important barrier for not using sunscreen is the cost of sunscreen (15), while providing free sunscreen has been reported as an effective intervention for promoting sunscreen use (21).

Apart from which prevention strategy is used, assessing the effect of such strategies in occupational circumstances is a challenge (21). Stratum corneum (SC) biomarkers showed to be promising markers to assess the internal UVR dose and immune response in experimental settings (22, 23). These are including *cis*-urocanic acid (cUCA), which is a sensitive, non-invasive marker of the internal UVB dose. However, its feasibility for assessing the UV-dose after chronic UVR exposure has not been investigated yet. The use of immunological SC markers—although less sensitive than cUCA—showed good possibilities to be suitable for detecting response at higher and/or repetitive UVR exposure.

We set up an intervention study focused on stimulating sunscreen use among outdoor construction workers, described in our previously published protocol (24). Unfortunately, due to the COVID-19 pandemic and the restrictive measures that were introduced, we were not able to perform the planned intervention study. Instead, we conducted a pilot study in which parts of the intervention study were carried out on a smaller scale (25, 26). For this pilot study, we followed the elements of study design reported by Blatch-Jones et al. (27) and adapted from Arain et al. (28). Elements investigated in this study were: recruitment, (loss to) follow-up, outcome measures, data collection, and the acceptability of the intervention. The main objective of this pilot study was to investigate the feasibility of these method- and intervention-related elements of the future intervention trial based on providing sunscreen and assessing sunscreen use on group level (monitoring usage) and individual level (SC biomarkers of UVR). We addressed the following research questions: what is the acceptability and feasibility of an intervention focused on providing sunscreen at the workplace? And what is the feasibility of collecting SC biomarkers of UVR exposure at the workplace?

## Materials and Methods

### Design and Setting

We conducted a pilot study in which we investigated the acceptability and feasibility of an intervention focused on providing sunscreen at the workplace (part 1). Secondly, we assessed the feasibility of collecting SC biomarkers of UVR exposure at the workplace (part 2). The duration of the study was 16 weeks (May-August 2021), and the setting was a construction site in a northern province of the Netherlands. Measurements consisted of two (cross-sectional) questionnaires, interviews with managers (part 1), and biochemical analyses of SC biomarkers of UVR exposure (part 2). The study protocol followed the principles of the Declaration of Helsinki (2013) and was approved by the Medical Ethics Committee of the Academic Medical Center, Amsterdam, the Netherlands.

### Participants and Recruitment

#### Part 1: Sunscreen Use

Participants were construction workers, engaged in outdoor work activities, and aged ≥18 years. The construction company (main contractor) was selected by the researchers because they offered frequent outdoor work tasks and therefore had a potentially high number of eligible participants. The eligible construction workers worked for several subcontractors hired by the main contractor. Work tasks consisted of scaffolding, fiber installation, paving, crane operation, and other construction work. The construction workers were recruited at the construction site by the researchers, and were informed on the study protocol in both oral and written form. Construction workers fulfilling the inclusion criteria were enrolled in the study and written informed consent was obtained.

#### Part 2: UV-Biomarkers

Fifteen indoor workers (IW) and fifteen OW were recruited at the construction site by the researchers. Participants either had a work task indoors (office workers) or a work task outdoors (construction workers). Participants were included on a first come, first serve basis, and inclusion was independent from their participation in the other part of this study. All participants had Fitzpatrick skin type 1, 2, 3 or 4 (29). Written informed consent was obtained.

### Study Procedures

**Figure 1:**
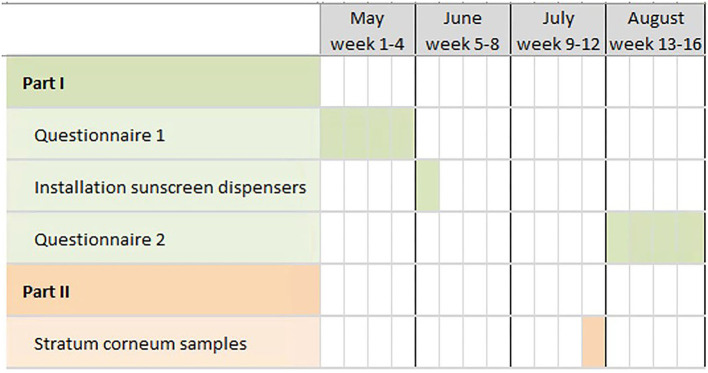
Gantt chart of the study procedures.

A Gantt chart of the study procedures is presented in Figure 1.

#### Part 1: Sunscreen Use

##### Questionnaires

The participants were asked to complete the first questionnaire at the start (T = 0; before the installation of the sunscreen dispensers), and the second questionnaire at the end of the study (T = 16 weeks; 2 months after installation of the sunscreen dispensers), this were cross-sectional and self-reported measurements. The questionnaire included socio-demographic questions about age, sex, and country of origin. Skin type was defined using the Fitzpatrick skin types (29). Work characteristics included work status as outdoor worker, job characteristics (e.g., job task), and number of years in current profession. Furthermore, there were questions about sun-related risk knowledge (e.g., “sun exposure is primary cause of skin cancer” or “must apply sunscreen even when it is overcast”), attitudes (e.g., “when the sun shines I spend as much time as possible outdoors” or “sunscreen use at work is important to me”), barriers for using sunscreen (e.g., “sunscreen use is easily fitted into my working day”), outside leisure-time spending (e.g., “I spend >3 hrs outside on my days off”), and UV-protective behaviors (e.g., use of sunscreen ever or in the previous month). In the second questionnaire (T = 16 weeks) an additional question about the number of sunburn episodes during the past 3 months was included, as well as questions about satisfaction with the provided sunscreen. Questions were assessed on a five-point Likert scale, or as correct/incorrect and yes/no answer options. The questionnaires are presented in Appendix I.

The questions in the questionnaires were based on a standardized set for measuring sun protection behavior in OW (30). A pilot version of the questionnaires was tested on four OW (not included in this study). Based on their feedback, some alterations were made to the questionnaires (i.e., better clarification of Fitzpatrick skin type). The questionnaires were available in seven languages (Bulgarian, Dutch, English, German, Hungarian, Polish, and Romanian) and were translated by a professional translation agency. The questionnaires were available on paper (for the non-technology oriented participants) and online. The online questionnaires could be completed using a smartphone or computer. LimeSurvey (Hamburg, Germany) was used as survey tool.

##### Sunscreen Dispensers

At the construction site, nine sunscreen dispensers were installed at readily accessible strategic places (e.g., the canteen, changing rooms, entrance etc.), 4 weeks after the first questionnaire was completed. Next to the dispensers, an informative poster was placed advising the OW to “apply sun cream”, presented in Appendix II. The dispensers were filled with sunscreen Stokoderm^®^ Sun Protect 50 PURE SPF 50 UV skin protection lotion for professional use. This product is a cosmetic product regulated by and complying with Regulation EC no. 1223/2009 (as amended) on Cosmetics Products. The main UV-protection ingredients are ethylhexyl salicylate, bis-ethylhexyloxyphenol methoxyphenyl triazine, butyl methoxydibenzoylmethane, octocrylene, and homosalate.

Initially, we planned to use electronic dispensers equipped with a Wi-Fi transmitter recording each application event. However, technical difficulties prohibited us to electronically register sunscreen applications. Instead, at the end of the study, we removed the cartridges from the dispensers and weighed them using an analytical balance in order to investigate in which location a dispenser was used most or least.

##### Interviews With Managers Regarding the Sunscreen Dispensers

At the end of the study, three managers working for the main contractor were interviewed individually regarding their experience with the sunscreen dispensers. This was done following predefined questions such as “What did you think of the sunscreen dispensers?”, “Where the dispensers located in practical places?” “Do you think this is a sufficient method to protect OW against the sun?” “Do you have tips to make this work better?”. The questions were asked as open questions, and more details were asked if needed. Each manager was interviewed for about 10 min.

#### Part 2: UV-Biomarkers

##### Stratum Corneum Biomarkers of UVR Exposure

SC samples were collected at T = 12 weeks. The SC was collected using adhesive tape strips, a minimally invasive, non-painful method which is extensively used in experimental studies (22, 31, 32). Adhesive tape discs (1.54 cm^2^, D-Squame; CuDerm, Dallas, TX, USA) were attached to the skin. Each tape was pressed on the skin for 5 s, using the thumb. The tape strips were removed gently with tweezers and stored in a closed vial at −80°C until analysis. SC samples (six tapes per sample location) were taken from skin sites exposed to the sun (i.e., the forehead) and a less exposed skin site (i.e., behind the ear).

##### Sample Analysis

Based on our previous studies (22, 23), the investigated markers of UVR exposure included *cis-* and *trans-*isomers of urocanic acid (UCA), and fifteen immunological markers: IL-18, IL-8, IL-33, IL-10, IL-1β, IL-1α, IL-1RA, MMP-9, VEGF, GM-CSF, MCP-4, MIP-1β, MIP-3α, CCL27, and CCL17.

##### Determination of UCA Isomers

The 3rd tape was used to determine *trans*-UCA (tUCA) and *cis*-UCA (cUCA). UCA isomers on the 3rd tape were extracted with 600 μl of Millipore water and subsequently analyzed by high-performance liquid chromatography (HPLC-UV), according to the method described in detail elsewhere (22, 33). The limit of detection is 0.14 μmol L^−1^, and the lower limit of quantitation is 0.45 μmol L^−1^.

##### Analysis of Immunological Markers

Extraction of immunological markers and soluble proteins from the 4th and 5th tape was performed as described before (22). In short, 1.2 mL phosphate-buffered saline (Merck, Darmstadt, Germany) with 0.005% Tween 20 (Sigma-Aldrich, Zwijndrecht, the Netherlands) was added to the cryo-vial containing the 4th tape. An ultrasound bath (Branson 5800, the Netherlands) was used for extracting immunological markers and soluble proteins. The extract from the 4th tape was transferred to the cryo-vial containing the 5th tape, repeating the procedure. Extract aliquots of 300 μL were distributed in micronic-vials and stored at −80°C until further analysis.

Concentrations of the fifteen immunological markers were determined using MESO QuickPlex SQ 120 (MSD, Rockville, MA, USA) according to the manufacturer's instructions. To correct for the variable amount of SC on each tape, the concentration of immunological markers was normalized by protein content, which was determined using Pierce Micro BCA Protein Assay Kit (Thermo Fischer Scientific, Rockford, IL, USA).

### Statistical Analysis

#### Part 1: Sunscreen Use

Data analyses for the questionnaires were performed with IBM SPSS Statistics for Windows, Version 26.0 (IBM Corp. Armonk, NY, USA). Answers were assessed on five-point Likert scales (disagree wholeheartedly, disagree, do not agree/do not disagree, agree, agree wholeheartedly; or never, rarely, sometimes, often, always), or correct/incorrect and yes/no answer options. Data analyses included counts with percentages and other descriptive statistics (median, IQR).

#### Part 2: UV-Biomarkers

Data analyses for the SC samples were performed with GraphPad Prism 8 software (GraphPad Software, San Diego, CA, USA). Data distribution was assessed with the Shapiro-Wilk normality test. Mann-Whitney *U* and Wilcoxon ranking tests were used for comparing the levels of immunological markers and cUCA between and within both groups (i.e., OW and IW), respectively. The relative amount of cUCA (*cis*-UCA/(*cis*-UCA+*trans*-UCA)) represents the proportion of initially present tUCA that is transformed to cUCA. Two-sided *p*-values of <0.05 were considered to be statistically significant. Data are presented as median with interquartile range (IQR) when non-normally distributed, or as mean values ± standard error of the mean (SEM) when distributed normally.

## Results

### Part 1: Sunscreen Use

After recruitment, 67 outdoor construction workers were eligible for inclusion in this study and were invited to complete the questionnaires. The first questionnaire was completed by 27 participants (loss to follow-up 60%) and the second one by 17 participants (loss to follow-up 75%). Background characteristics are presented in Table 1. Median age (IQR) of the participants was 33 years (22, 24–41)—first questionnaire—and 35 years (22, 29–42)—second questionnaire—. They mostly had skin type III, and a majority of the participants were male and smokers. Approximately 20% of the participants worked in construction for over 20 years, and the majority worked outside for >4 h per day.

**Table 1:** Background characteristics of outdoor workers.

	**Questionnaire 1** **(T = 0 weeks) *n* = 27**	**Questionnaire 2** **(T = 16 weeks) *n* = 17**
Age (years ± SD)	34 ± 9.7	36 ± 8.8
Sex (males, *n*, %)	25 (93%)	15 (88%)
**Smoking (*****n**,* **%)**		
Never	8 (29%)	4 (24%)
Quit	2 (7%)	2 (12%)
Yes	17 (63%)	11 (65%)
***packs/day***		
*0.25*	1 (4%)	
*0.5*	6 (22%)	4 (24%)
*1*	9 (33%)	7 (41%)
*2*	1 (4%)	
**Skin type (*****n**,* **%)**		
I	2 (7%)	1 (6%)
II	2 (7%)	5 (29%)
III	19 (70%)	8 (47%)
IV	4 (15%)	3 (18%)
**Country of birth (*****n**,* **%)**		
Belgium	1 (4%)	
Bosnia and Herzegovina	3 (11%)	
Brazil	1 (4%)	
Bulgaria	3 (11%)	2 (12%)
Croatia	1 (4%)	1 (6%)
Hungary	1 (4%)	
Ireland	5 (19%)	2 (12%)
Lithuania	1 (4%)	1 (6%)
Poland	3 (11%)	
Romania	3 (11%)	5 (30%)
Spain	1 (4%)	
The Netherlands	1 (4%)	4 (24%)
United Kingdom	3 (11%)	2 (12%)
**Job as construction worker**		
0–1 year	5 (19%)	2 (12%)
2–5 years	9 (33%)	5 (29%)
6–10 years	7 (26%)	1 (6%)
11–20 years	2 (7%)	5 (30%)
>20 years	4 (24%)	3 (18%)
**Outdoor on workday (*****n**,* **%)**		
0–1 h	5 (19%)	1 (6%)
2–4 h	7 (26%)	5 (29%)
>4 h	15 (56%)	11 (65%)
**Work task outdoor**		
Scaffolding fitter	5 (19%)	1 (6%)
Fiber installation	8 (30%)	2 12%)
Paving	2 (7%)	3 (18%)
Crane operator	2 (7%)	
Other	10 (37%)	11 (65%)

#### Sunscreen Use Behavior

Table 2 shows the results of sunscreen use behavior of the participants. Before installation of the sunscreen dispensers, 19% (*n* = 5) of the OW never considered using sunscreen, this percentage was 18% (*n* = 3) after the dispenser installation. At baseline, 30% (*n* = 8) of the OW “sometimes” applied sunscreen in the past month, this was 41% (*n* = 7) at the end of the study. “Never” applying sunscreen in the past month was 44% (*n* = 12) in the first questionnaire, and 35% (*n* = 6) in the second. Furthermore, the majority of the participants reported that sunscreen provided by the employer (59%, *n* = 16 and 53%, *n* = 9, respectively), and sunscreen as protection against skin cancer (85%, *n* = 23 and 71%, *n* = 12, respectively) were encouraging reasons for using sunscreen at work.

**Table 2:** Sunscreen use behavior.

**Question**	**Answers**	**Questionnaire 1** **(T = 0 weeks) *n* = 27**	**Questionnaire 2** **(T = 16 weeks) *n* = 17**
Sunscreen use	Never considered using	5 (19%)	3 (18%)
	Considered it, not decided	5 (19%)	4 (24%)
	Not using	3 (11%)	–
	Will use	2 (7%)	4 (24%)
	Already using	11 (41%)	6 (35%)
Sunscreen	Never	12 (44%)	6 (35%)
application past	Rarely	4 (15%	2 (12%)
month	Sometimes	8 (30%)	7 (41%)
	Often	1 (4%)	2 (12%)
	Always	1 (4%)	–
Sunscreen	0	9 (33%)	6 (35%)
applications per	1	12 (44%)	7 (41%)
day	2	4 (15%)	2 (12%)
	3	1 (4%)	1 (6%)
	≥4		1 (6%)
Sunscreen application times	Morning before work	15 (56%)	8 (47%)
(multiple answers	Coffee break	4 (15%)	7 (41%)
possible)	Lunch	8 (30%)	7 (41%)
Encouraging sunscreen use	Provided by employer	16 (59%)	9 (53%)
(only “yes”)	Employer regularly emphasizes its importance	13 (48%)	8 (47%)
	Colleague also use sunscreen	14 (52%)	6 (35%)
	Protecting against skin cancer	23 (85%)	12 (71%)
Not considering sunscreen use necessary	When the sun shines I work in the shade	12 (44%)	8 (47%)
because: (only“yes”)	I use protective clothing	16 (59%)	10 (59%)
	I like tan skin	13 (48%)	8 (47%)

#### Knowledge of Sun Risks and Behavior in the Sun

**Figure 2:**
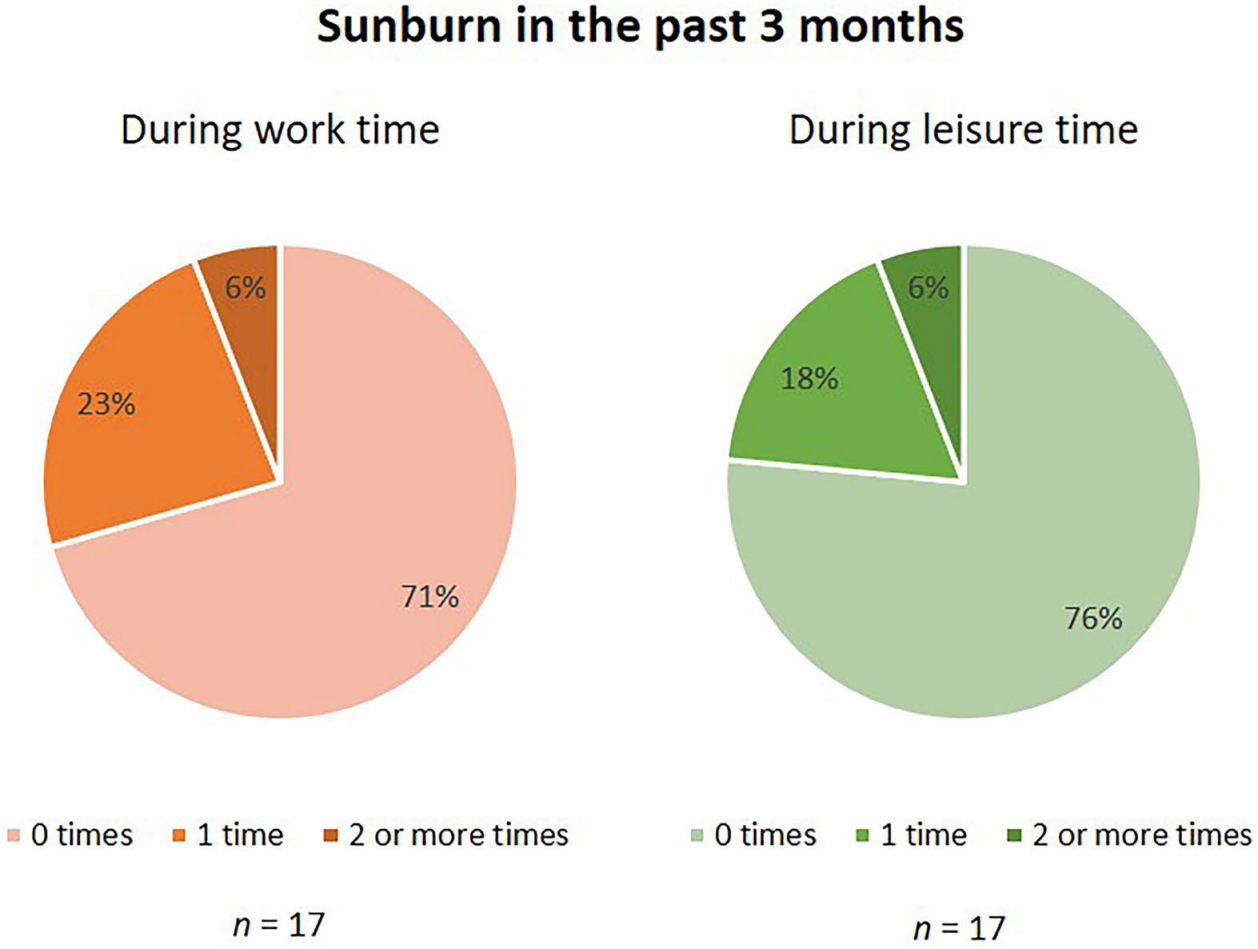
Sunburn in the past 3 months. Numbers are percentages (%). Assessed in questionnaire 2 (*n* = 17).

Table 3 presents the knowledge of OW regarding sun risks. At baseline, the majority of OW (82%, *n* = 22) knew that sun exposure is the primary cause of skin cancer, this percentage was 100% (*n* = 17) after the installation of the dispensers. Furthermore, almost every OW knew that sunscreen is still important even if you have a tanned skin (96%, *n* = 26 and 88%, *n* = 15, respectively). For approximately two thirds (63%, *n* = 17 and 65%, *n* = 11, respectively) of the OW, the use of sunscreen at work is of importance, and for 67% (*n* = 18) and 82% (*n* = 14), respectively, it is easily fitted into the working day. In Figure 2 is shown that the majority of OW has not had a sunburn in the previous 3 months during work (71%), as well as during leisure time (77%).

**Table 3:** Knowledge of sun risks and behavior in the sun.

**Question**	**Answers**	**Questionnaire 1 (T = 0 weeks) *n* = 27**	**Questionnaire 2 (T = 16 weeks) *n* = 17**
Must apply	Correct	15 (56%)	13 (77%)
sunscreen even when it is overcast	Incorrect	12 (44%)	4 (24%)
Sun exposure is	Correct	22 (82%)	17 (100%)
the primary cause of skin cancer	Incorrect	5 (19%)	
If I have tan skin I	Correct	1 (4%)	2 (12%)
no longer need to apply sunscreen	Incorrect	26 (96%)	15 (88%)
When the sun shines: (only “yes”)	I spend as much time as possible outdoors	14 (52%)	9 (53%)
	I always use sun protection	15 (56%)	9 (53%)
	I seek shelter in the shade or stay indoors	15 (56%)	8 (47%)
	I spend >3 h outdoors on my days off work	17 (63%)	12 (71%)
Sunscreen use at	Is important to me	17 (63%)	11 (65%)
work (only “yes”)	Is easily fitted into my working day	18 (67%)	14 (82%)
	Is something I do before I start working outdoors	16 (59%)	8 (47%)

#### Facilitation of Sunscreen Dispensers at Worksite

OW were satisfied regarding the sunscreen dispensers placed on the worksite: 65% (*n* = 11) agreed that the dispensers were easy to use, and 59% (*n* = 10) agreed that the dispensers were located in practical spots. Sunscreen was not seen as a nuisance during work by 47% (*n* = 8), and 53% (*n* = 9) found the sunscreen easy to apply and not sticky. Information on posters helped 53% (*n* = 9) of OW to use sunscreen, and a majority of OW (65%, *n* = 11) would recommend the dispensers and posters to colleagues. When used, sunscreen was mostly applied to the face (94%, *n* = 16), and in lesser amounts to the arms, legs and chest/stomach/back. See Appendix III for Figure.

#### Sunscreen Consumption

Nine sunscreen dispensers were installed at strategic places at the construction site for 14 weeks, i.e., 71 work days. The dispenser used most was placed at the exit of the canteen, second at the entrance of the canteen and third was the dispenser placed at the entrance of the changing rooms. Least used dispensers were placed next to the outside smoking area and in the scaffolder's office.

#### Interviews With Managers Regarding Sunscreen Dispensers

The interviews with the managers revealed a variety of experiences. Overall, the conclusion was that the sunscreen dispensers were a positive asset to the construction site and the dispensers as well as the sunscreen were easy in use. However, differences were found in their opinions whether dispensers are the optimal way of offering sunscreen. Also, the scaffolder's manager said “The scaffolders are wearing a lot of personal protective equipment (including mouth masks), so there is not much skin left to apply sunscreen on,” suggesting that scaffolders are not the optimal pilot group for stimulating sunscreen use. Lastly, the sunscreen dispensers were very similar to the hand disinfection gel dispensers, resulting in some OW mistakenly applying sunscreen when they initially wanted to use hand disinfection gel.

### Part 2: UV-Biomarkers

#### Urocanic Acid Isomers

**Figure 3:**
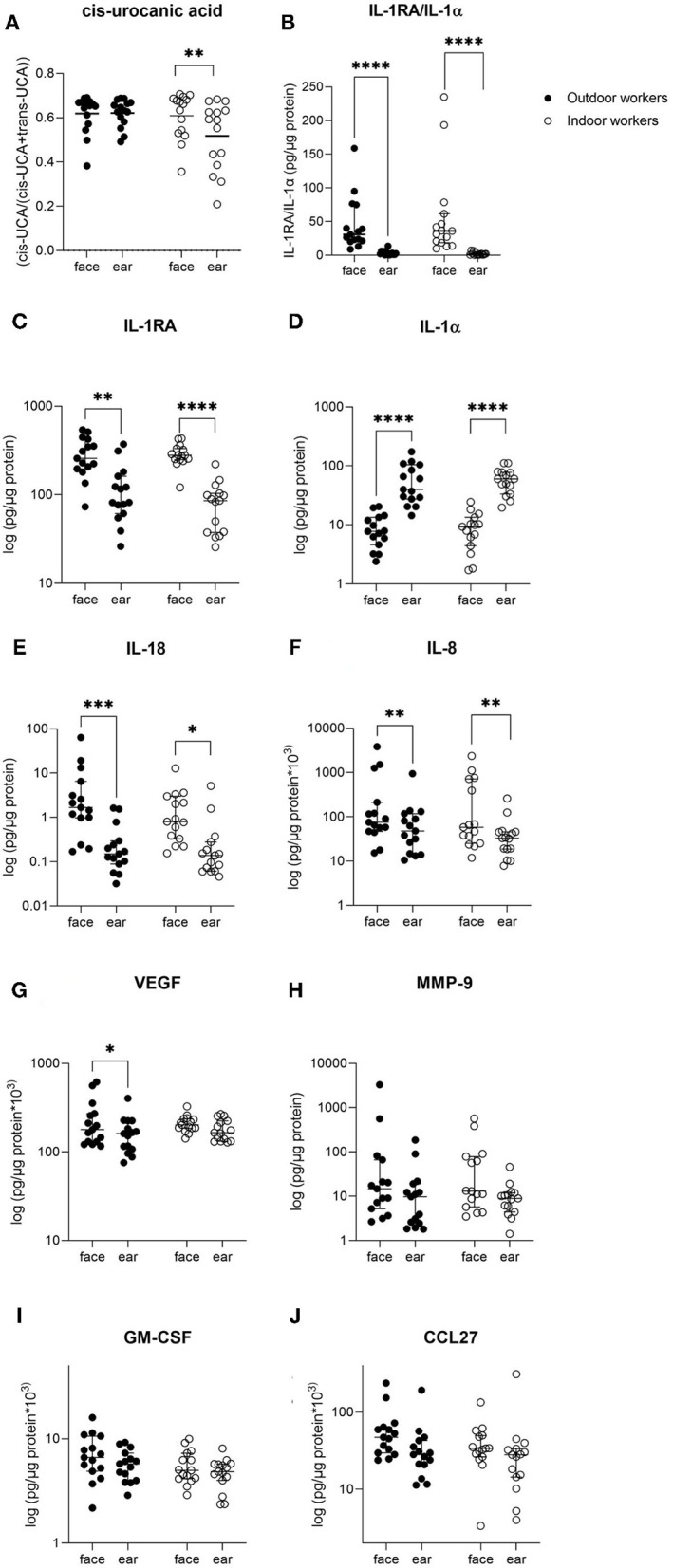
Overview of stratum corneum concentrations of several markers measured in outdoor and indoor workers. Data are presented as median with interquartile ranges. Differences in concentrations between both groups were tested using Mann-Whitney *U*-test. Differences in concentrations between sample locations (forehead and ear) were tested using Wilcoxon ranking tests. *p < 0.05, **p < 0.01, ***p < 0.001, ****p < 0.0001.

Figure 3A shows that the relative amount of cUCA was not significantly different between OW and IW. However, there was a significant difference between the levels of cUCA measured in IW between the forehead and behind the ear. The concentration of cUCA in OW were similar for the forehead and behind the ear, both reaching a level of 63%.

#### Immunological Markers

Figures 3B–J shows the levels of various immunological markers in OW and IW, measured in the SC sampled from the forehead and behind the ear. None of the fifteen included immunological markers (not all presented) showed significantly different levels between OW and IW. Comparison of the two investigated skin sites revealed that concentrations of IL-18, IL-8, IL-1α, IL-1RA and the ratio of IL-1RA/IL-1α were significantly different between the sun-exposed and less exposed sample locations (i.e., forehead and behind the ear) for OW as well as IW. VEGF was only significantly different between the forehead and ear in OW (Figure 3G).

## Discussion

This pilot study revealed low sunscreen use among OW, despite providing sunscreen at the workplace, overall satisfaction with the sunscreen, and sufficient awareness of the risks of UVR exposure. The collection of SC samples at the workplace is feasible, and several UV-biomarkers showed to be promising in assessing UVR exposure. Several method- and intervention-related elements of a future intervention trial were investigated, the elements recruitment and (loss to) follow-up need more attention since the participation rate was low and the loss to follow-up high.

### Part 1: Sunscreen Use

This pilot study revealed low sunscreen use among OW, although the number of OW who reported never using sunscreen in the previous month somewhat decreased (44.4–35.3%) after placement of the sunscreen dispensers. Low usage of sunscreen is in accordance with several other studies. In a study of Zink et al. (34), almost half of the participants reported that they seldom or never used sunscreen at work. Grandahl et al. (35) found that 33% of the OW rarely or never used any type of sun protection at work. Peters et al. (36) found that sunscreen use was low especially in male OW. Consistently, a systematic review by Reinau et al. (37) showed that OW have poor protection against UVR exposure at work, concluding that a vast majority of agricultural and construction workers rarely or never applied sunscreen at work. A possible reason for the low usage of sunscreen by OW in the present study might be caused by the rainy weather during the study period as compared with the average number of sunny days in the same period in previous years (38). Furthermore, approximately two thirds of the participants in this study reported that they wear protective clothing during work. According to the safety manager, the workers are obliged to wear hard hats, long-sleeved shirts, and—because of the pandemic—a face mask, which left the exposed skin area small. Behavior concerning the use of protective clothing among OW is largely dependent on the safety requirements of the employer and differences between studies are large. For example, Peters et al. (36) found that 82% of the OW wore protective clothing in the form of long-sleeved shirts while a systematic review of Reinau et al. (37), reports percentages of OW wearing protective clothing between 20 and 50%. Another factor that might have an influence on sunscreen use, is the time spent outside: in our study approximately one third of the OW worked outside for <4 h per day. The combination of wearing protective clothing and spending less time outside might have contributed to the low occurrence of sunburns in our study. The majority of OW never had a sunburn during work in the past 3 months, while other studies reported the opposite: 50–80% of the OW had a history of sunburn at work (35, 37).

We asked the OW for their knowledge on the risk of skin cancer, believes on sunscreen use, and protection measures as this information might be useful for setting up an intervention. A vast majority of OW in our study was aware that UVR exposure is the primary cause of skin cancer, that they have to apply sunscreen when it is overcast, and that a tanned skin is not a reason to stop using sunscreen. This is consistent with other studies where OW had good knowledge of skin cancer facts (34, 37). It is encouraging that the majority of OW in our study reported that sunscreen use is easily fitted into the work day, whereas Zink et al. (34) found that 50% of the OW had difficulties implementing these measures into their routine. Sunscreen provided by the employer at various easily accessible places at the workplace encourages sunscreen use, reported OW in this study. Furthermore, they rated the sunscreen as easy to apply which is important information for future intervention studies.

### Part 2: UV-Biomarkers

Collection of SC samples at the workplace showed to be feasible. Duration of sampling was ~5 min per participant and the participants did not experience any discomfort. As expected, several immunological markers showed a significant difference between the two investigated skin sites. The differences in levels of immunological markers between OW and IW was not significant, however, several markers such as IL-18, IL-8, CCL27, and GM-CSF showed a pattern of higher values in OW compared with IW. It has to be noted that these IW were not the ideal control group as some of them just returned from holiday and likely had high exposure to UVR. In future intervention studies where intervention (with sunscreen) and control groups (without sunscreen) will be compared at different time points during the study, a larger difference in levels of immunological biomarkers may be expected.

cUCA levels in IW were higher in the sun-exposed skin site compared to the less exposed skin site, but in OW cUCA reached a saturation level of approximately 60% in both investigated skin sites. cUCA is formed from tUCA upon exposure to UVB in a dose-dependent manner until reaching a photo stationary state at ~60–70% of total UCA (39). Whether cUCA is suitable to assess the effect of sunscreen use will largely depend on the degree of reduction in UVR exposure (i.e., UVR exposure under the cUCA saturation level). It should be kept in mind that the relative amount of cUCA in the unprotected and chronic UVR exposed skin will likely reach saturation level and only a qualitative measure of the difference between two groups can be obtained.

The finding that immunological markers differ in levels in the two differently exposed skin sites is encouraging and confirms our previous data (22, 23) that they might be particularly useful to assess chronic exposure to UVR. Furthermore, they are also important for assessing the immune response in the skin which plays an important role in UVR mediated damage (40) and might even occur before visible changes (erythema of the skin) are seen. Several immunological markers investigated in this study seem to be suitable to assess those effects of UVR exposure (41–44).

### Lessons Learned From This Pilot Study: Strengths and Limitations

In this pilot study we investigated the feasibility of several elements (i.e., recruitment, (loss to) follow-up, outcome measures, data collection, and the acceptability of the intervention) which revealed some challenges that need to be addressed. First, the number of OW that was willing to participate in this study was much lower than we expected. After recruitment, 67 construction workers were eligible for participation in this study, but only *n* = 27 participants completed the first questionnaire and even less (*n* = 17) completed the second one. In a post-study interview, the site managers recommended to communicate the relevance of sun-safety behavior clearer to the OW by using other communication channels. As suggested by the site managers, visual aids, such as an UV-lamp, showing the skin damage already present in their faces, or offering skin checks by trained physicians for signs of sun damage or skin cancer might be more effective to motivate OW to participate in the study. Second, the selection of eligible outdoor construction workers for participation in a study stimulating sunscreen use has to be accurate. In this study we also included scaffolders who wore substantial amounts of personal protective equipment, which left almost no skin parts free for sunscreen application. Consequently, we probably have a lack of urgency for using sunscreen during worktime in our pilot group. In future studies, a longer study period would compensate for fluctuations in outdoor work tasks at a construction site. Furthermore, construction workers who wear lesser amounts of personal protective equipment (i.e., more exposed skin area) will be included. Third, we encountered a language barrier since the majority of the construction workers did not speak Dutch or English (despite English being the official language at this construction site), making it difficult to verbally inform or answer questions. Having a “workplace champion” to serve as a contact person at the study site during the project with good command of English and/or Dutch, and the mother tongue of the group of OW would improve verbal communication, since the number of foreign nationals working in construction is increasing (45). Fourth, it is recommended to also collect the opinions of several stakeholders—including the OW themselves—on what is needed to make OW more aware of sun-safety behavior during worktime with, for instance, a focus group.

Fifth, our study period of 16 weeks might have been too short to achieve behavior change. It is reported that longer duration of the study probably has more chance to lead to change in behavior (46). Particularly important to mention for this pilot study is the rotating system that was used at the construction site: OW worked for 2 months followed by 1 month time off. This might have influenced on their commitment in participating in a 4-month study. For future intervention studies it is recommended to select OW who remain at the same construction site for a longer time. Sixth and last, the low usage of sunscreen indicates that providing sunscreen at easily accessible places at the workplace as standalone intervention is not enough to increase sunscreen use among OW substantially. The message to use sunscreen during work should be repeated continuously. For example, by implementing structured feedback on sunscreen use at that specific workplace in order to motivate and improve compliance (47). This message should be produced in a colorful and illustrative format which helps to transmit it more effectively (45, 48). Lack of awareness on the risks of UVR exposure, common misbelieves such as “people with a tanned skin are not at risk for skin cancer”, and concerns regarding the interference of sunscreen with work activities were not identified as barriers among participants in this study. Additionally, we removed common barriers such as availability, accessibility, and costs of sunscreen (9, 20, 21) by installing sunscreen dispensers at strategic places at the construction site.

In this pilot study, the installation of sunscreen dispensers at strategic places at the workplace encouraged sunscreen use among OW. Regarding the acceptability of the intervention, OW reported that they were satisfied with the dispensers as well as the sunscreen, and that it did not interfere with their work tasks. This is an important finding, which means that this intervention can be continued in future studies. Furthermore, the questionnaires were a feasible tool when presented as an online platform, so participants were able to complete the questionnaire on their smartphone or computer. Also, the use of QR codes (quick response) appeared to be feasible. Additionally, it is important to make the questionnaires available in several languages, in order to avoid a language barrier. A limitation, as always for self-completed questionnaires, is that a recall bias or social desirable answers cannot be excluded. Lastly, with the UV-biomarkers we have found a feasible tool to use at the workplace for assessing the internal UVR dose (i.e., UV-dose absorbed by the skin). Collection of SC samples is easy, simple, and painless for the participant. For future intervention studies, we recommend to also measure the external UVR exposure using personal dosimeters to get more insight in the UV-exposure pattern during the work shift and different work tasks.

In summary, this pilot study revealed that three of the investigated elements (i.e., outcome measure, data collection, and the acceptability of the intervention) are feasible. Providing sunscreen dispensers at the workplace seems feasible, but not as standalone intervention for stimulating sunscreen use. Collecting SC biomarkers of UVR exposure at the workplace is feasible and the markers showed to be promising in assessing UVR exposure. However, the elements recruitment and (loss to) follow-up need more attention since the participation rate was low and the loss to follow-up high. This poses a challenge for future intervention studies.

## Data Availability Statement

All relevant data is contained within the article: The original contributions presented in the study are included in the article/Supplementary Files, further inquiries can be directed to the corresponding author.

## Ethics Statement

The studies involving human participants were reviewed and approved by Medical Ethics Committee of the Academic Medical Center, Amsterdam, the Netherlands. The participants provided their written informed consent to participate in this study.

## Author Contributions

AK was largely involved in the conception, design, and operational management of the study, was responsible for conducting the study and the (biochemical) assessments during the study, and prepared the manuscript. SK has expertise in skin bioengineering methods for the assessment of stratum corneum samples, was involved in the conception, design and supervision of the trial, and was involved in drafting the manuscript together with AK. HM brings expertise in occupational health, pragmatic research, and translating research findings into policy and was involved in the conception and design of the trial, and drafting the manuscript together with AK. TR brings in expertise in dermatology, pragmatic research, and critically reviewed the manuscript. CH brings expertise in occupational health, pragmatic research, and critically reviewed the manuscript. All authors read and approved the final manuscript.

## Conflict of Interest

The authors declare that the research was conducted in the absence of any commercial or financial relationships that could be construed as a potential conflict of interest.

## Publisher's Note

All claims expressed in this article are solely those of the authors and do not necessarily represent those of their affiliated organizations, or those of the publisher, the editors and the reviewers. Any product that may be evaluated in this article, or claim that may be made by its manufacturer, is not guaranteed or endorsed by the publisher.
