# Supplementary material for: Stimulating Sunscreen Use Among Outdoor Construction Workers: A Pilot Study
Source: Front Public Health. 2022 Apr 1;10:857553. doi: 10.3389/fpubh.2022.857553 (PMC9010717; doi:10.3389/fpubh.2022.857553)
Supplement: Supplementary file 1 [file Data_Sheet_1.PDF]

<u>Appendix I - Questionnaires</u>

<u>Sun protection at work</u>

Questionnaire (commencement of study)

ID:

General background

Dear participant,

This is a questionnaire containing questions regarding working outdoors and the use of sunscreen at work. You have received this questionnaire because you are participating in the study "Sun protection at work". Since in this study we are interested in hearing your opinion, we ask you to complete the questionnaire.
There are no correct or incorrect answers. For this study it is important that you answer <u>all</u> of the questions. It will take approximately five minutes to complete the questionnaire.

Confidentiality
The responses you give to the answers will be treated confidentially. Your employer will <u>not</u> be given access to your personal replies. You will receive another questionnaire at the end of the study.

We kindly request you to hand the completed questionnaire back to the investigator.
If you have questions regarding the questionnaire, please contact the investigator or send an email to: a.j.keurentjes@amsterdamumc.nl

Instructions for completion

You can answer the open questions with numbers or text. Answer the multiple-choice questions by placing a cross in the box that best corresponds with your answer: ☒.

If you accidentally placed a cross in the wrong box, place a cross in the correct box and then circle the correct box: ☒

General questions

1. What is your gender? ❑ male ❑ female

2. What is your age? ______ years

3. What is your country of birth? ________________________________

4. What is your natural hair color? ________________________________
   (if grey, what was your hair color before you went grey?)

5. What color are your eyes?
   ____

6. Indicate which answer best describes your skin:
   - ❑ Fair skin, red hair. Burns easily, does not tan.
   - ❑ Fair skin, blonde hair. Burns easily, tans slowly.
   - ❑ Light colored skin, brown hair. Rarely burns, tans easily.
   - ❑ Colored skin, dark hair. Almost never burns, tans very well.
   - ❑ Asian skin type, handles sun very well.
   - ❑ Black skin, handles sun very well.

7. Do you smoke?
   ❑ yes, _______ pack(s) a day
   ❑ no, I quit ________ ago
   ❑ no, have never smoked

8. How many years have you been working in construction? ______ years

9. What is your most important outdoor task? ________________________________
   (for example asphalt laying, paving, etc.)

10. How many hours do you work outdoors on an average working day?
    ❑ 0-1 hour ❑ 2-4 hours
    ❑ 1-2 hours ❑ More than 4 hours

11. Date on which you completed this questionnaire:
    _____ /_____ /20_____ (day-month-year)

Questions about sunscreen

12. Which of the statements below best applies to you?
    - ❑ I have never considered using sunscreen
    - ❑ I have considered it, but do not yet know whether I will use sunscreen
    - ❑ I have decided **not** to use sunscreen
    - ❑ I have decided that I **will** use sunscreen
    - ❑ I already use sunscreen

13. In the past month I applied sunscreen every day:
    - ❑ Never
    - ❑ Rarely
    - ❑ Sometimes
    - ❑ Often
    - ❑ Always

14. How many times a day do you apply sunscreen on average?
    - ❑ 0
    - ❑ 1
    - ❑ 2
    - ❑ 3
    - ❑ 4 or more

15. I applied sunscreen at the following times: (give an answer for all 3 these times)

	Yes	No
In the morning before I start work	❑	❑
During the morning coffee break	❑	❑
At lunch	❑	❑

**I feel encouraged to use sunscreen:**	**Yes**	**No**
16. … if my employer provides it		
17. … if my employer regularly emphasizes its importance		
18. … if my colleagues also use sunscreen		
19. … as protection against skin cancer		
**I do not consider it necessary to use sunscreen because:**	**Yes**	**No**
20. … when the sun shines I work in the shade		
21. … I use protective clothing		
22. … I like tan skin		
**Questions about the sun**	**Correct**	**Incorrect**

23. I must apply sunscreen even when it is overcast		
24. Exposure to the sun is the primary cause of skin cancer		
25. If I already have tan skin I no longer need to apply sunscreen		
**When the sun shines:**	**Yes**	**No**
26. … I spend as much time as possible outdoors		
27. … I always use protection against the sun		
28. … I seek shelter in the shade or stay indoors		
29. … I spend more than 3 hours outdoors on my days off work		
**The use of sunscreen at work:**	**Yes**	**No**
30. … is important to me		
31. … can easily be fitted into my working day		
32. … is something I do before I start working outdoors		

Suggestions for improvement:

……

……

……

……

Thanks for completing this questionnaire!

Sun protection at work

Questionnaire (end of study)

ID:

General background

Dear participant,

This is a questionnaire containing questions regarding working outdoors and the use of sunscreen at work. You have received this questionnaire because you are participating in the study “Sun protection at work”. Since in this study we are interested in hearing your opinion, we ask you to complete the questionnaire.
There are no correct or incorrect answers. For this study it is important that you answer all of the questions. It will take approximately five minutes to complete the questionnaire.

Confidentiality
The responses you give to the answers will be treated confidentially. Your employer will not be given access to your personal replies. This is the last questionnaire for this study.

We kindly request you to hand the completed questionnaire back to the investigator.
If you have questions regarding the questionnaire, please contact the investigator or send an email to: a.j.keurentjes@amsterdamumc.nl

Instructions for completion

You can answer the open questions with numbers or text. Answer the multiple-choice questions by placing a cross in the box that best corresponds with your answer: ☒.

If you accidentally placed a cross in the wrong box, place a cross in the correct box and then circle the correct box: ☒

Questions about sunscreen

1. Which of the statements below best applies to you?
   - ❑ I have never considered using sunscreen
   - ❑ I have considered it, but do not yet know whether I will use sunscreen
   - ❑ I have decided **not** to use sunscreen
   - ❑ I have decided that I **will** use sunscreen
   - ❑ I already use sunscreen

2. In the past month I applied sunscreen every day:
   - ❑ Never
   - ❑ Sometimes
   - ❑ Regularly
   - ❑ Often
   - ❑ Always

3. How many times a day do you apply sunscreen on average?
   - ❑ 0
   - ❑ 1
   - ❑ 2
   - ❑ 3
   - ❑ 4 or more

4. I applied sunscreen at the following times: (give an answer for all 3 these times)

	Yes	No
In the morning before I start work	❑	❑
During the morning coffee break	❑	❑
At lunch	❑	❑

**I feel encouraged to use sunscreen:**	**Yes**	**No**
5. … if my employer provides it		
6. … if my employer regularly emphasizes its importance		
7. … if my colleagues also use sunscreen		
8. … as protection against skin cancer		
**I do not consider it necessary to use sunscreen because:**	**Yes**	**No**
9. … when the sun shines I work in the shade		
10. … I use protective clothing		
11. … I like tan skin		
**Questions about the sun**	**Correct**	**Incorrect**

12. I must apply sunscreen even when it is overcast		
13. Exposure to the sun is the primary cause of skin cancer		
14. If I already have tan skin I no longer need to apply sunscreen		
**When the sun shines:**	**Yes**	**No**
15. … I spend as much time as possible outdoors		
16. … I always use protection against the sun		
17. … I seek shelter in the shade or stay indoors		
18. … I spend more than 3 hours outdoors on my days off work		
**The use of sunscreen at work:**	**Yes**	**No**
19. … is important to me		
20. … can easily be fitted into my working day		
21. … is something I do before I start working outdoors		

22. How many times did you sustain a red OR painful sunburn during the past 3 months **during work**?
    - ❑ 0 times
    - ❑ 1 time
    - ❑ 2 times
    - ❑ More than 3 times

23. How many times did you sustain a red OR painful sunburn during the past 3 months **on a day off**?
    - ❑ 0 times
    - ❑ 1 time
    - ❑ 2 times
    - ❑ More than 3 times

Questions about the “Sun protection at work” study

	Disagree wholeheartedly	Disagree	Do not agree, do not disagree	Agree	Agree wholeheartedly
24. The dispensers are easy to use	1	2	3	4	5
25. The dispensers are located in a practical spot	1	2	3	4	5
26. The sunscreen is not a nuisance during my work	1	2	3	4	5
27. The sunscreen is easy to apply and is not sticky	1	2	3	4	5
28. The posters helped me use the sunscreen	1	2	3	4	5
29. I started using more sunscreen than I used to before the study	1	2	3	4	5
30. I would recommend the dispensers and posters to my colleagues	1	2	3	4	5

31. Which part(s) of your body do you apply sunscreen to when you do use sunscreen? (more than answer possible)
    - ❑ face
    - ❑ arms
    - ❑ legs
    - ❑ chest/stomach/back

Suggestions for improvement:

……

……

……

……

Thanks for completing this questionnaire!

Appendix II – Poster

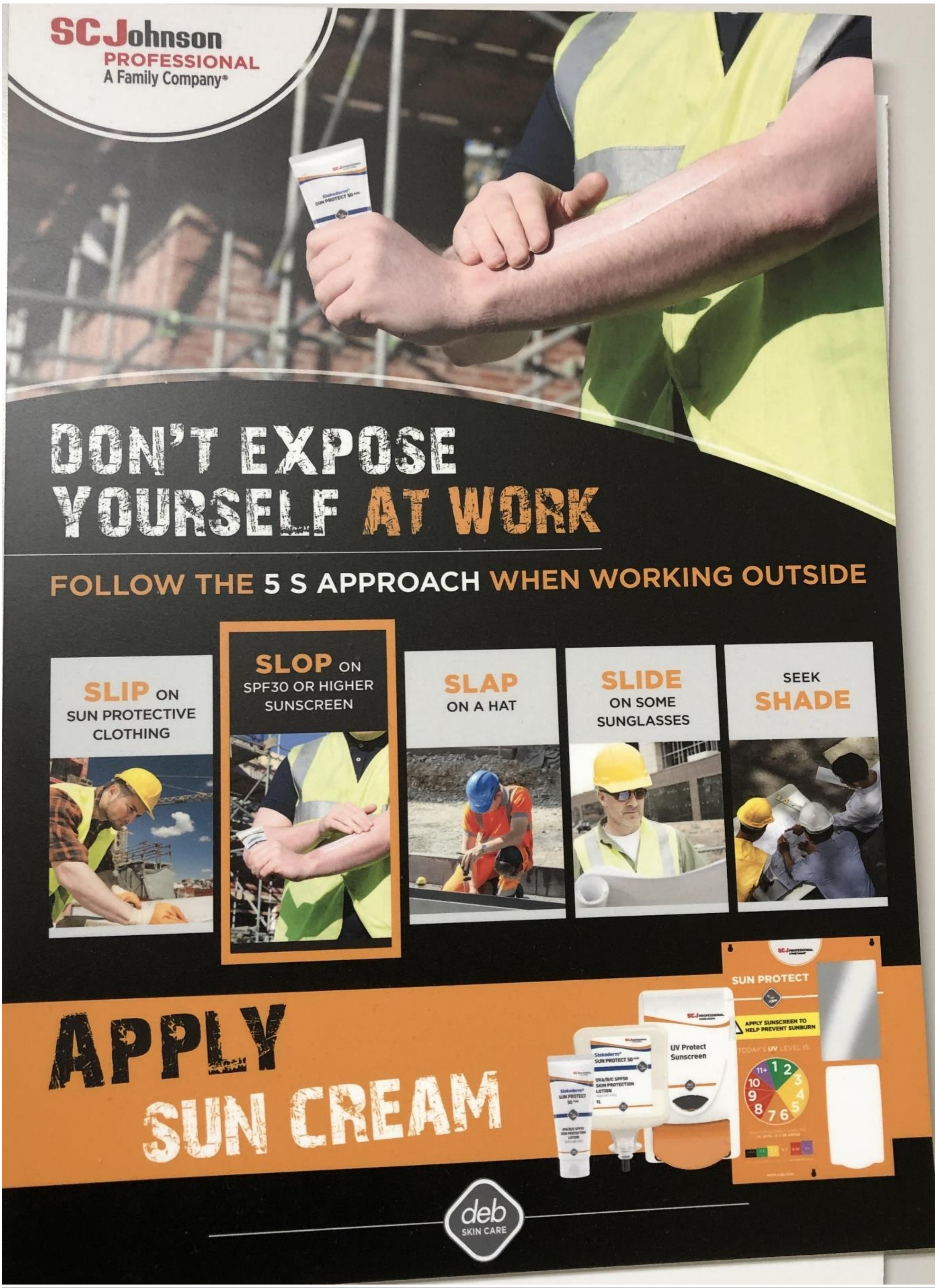

Appendix III – Figure

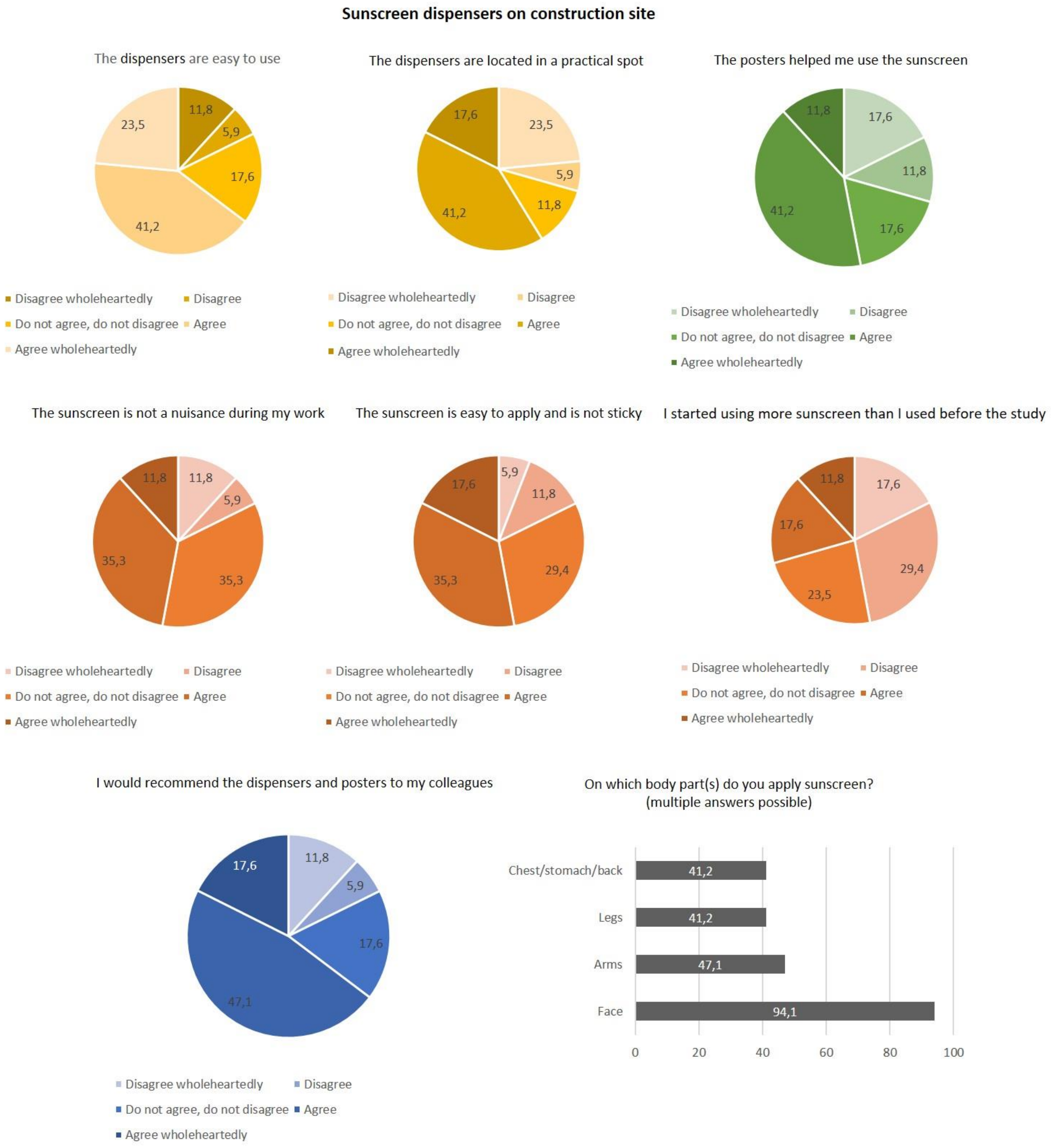

Figure - Sunscreen dispensers on construction site. Numbers are percentages (%). Assessed in questionnaire 2 (*n* = 17).
